# Agro-Morphological and Cytogenetic Characterization of *Hibiscus* Genetic Resources: Implications for Germplasm Conservation and Interspecific Breeding

**DOI:** 10.3390/plants15111633

**Published:** 2026-05-26

**Authors:** Yaqoob Sultan, Deen Mohammad Deepo, Ki-Byung Lim, Eglė Norkevičienė

**Affiliations:** 1 Department of Horticultural Science, Kyungpook National University, Daegu 41566, Republic of Korea; yaqoobsultan@knu.ac.kr; 2 Lithuanian Research Centre for Agriculture and Forestry, Instituto al. 1, Akademija, LT-58344 Kedainiai, Lithuania; egle.norkeviciene@lammc.lt; 3 Institute of Seed Technology, Sher-e-Bangla Agricultural University, Dhaka 1207, Bangladesh; 4 Institute of Agricultural Science and Technology, Kyungpook National University, Daegu 41566, Republic of Korea

**Keywords:** cytogenetics, chromosome number, fluorescence in situ hybridization (FISH), rDNA loci

## Abstract

Plant genetic resources are vital for crop improvement, ecological resilience, and agrobiodiversity conservation, making their characterization through morphological and cytogenetic methods essential for breeding and germplasm management. This study comparatively analyzed two herbaceous cultivars *Hibiscus moscheutos* cv. ‘Carousel Jolly Heart’ and cv. ‘Carousel Pink Passion’ and two woody cultivars, *Hibiscus syriacus* cv. ‘Sukim’ and cv. ‘Freedom’, to assess interspecific diversity and hybridization potential. Morphological assessments revealed notable differences in flower size and leaf shape between species, with ‘Carousel Pink Passion’ exhibiting the largest flower diameter (16.70 cm) and ‘Freedom’ exhibiting the smallest (10.20 cm). Chromosome analysis confirmed diploidy (2n = 38) in *H. moscheutos* and polyploidy (2n = 84) in *H. syriacus*, highlighting a fundamental genomic distinction between the two species. Fluorescence in situ hybridization (FISH) consistently identified two 5S rDNA loci across all cultivars; however, species-specific variation in 18S rDNA loci was detected with four loci in *H. syriacus* and six in *H. moscheutos*, suggesting divergent rDNA evolution and distinct genomic organization in the two species. Flow cytometry confirmed significant differences in nuclear DNA content corresponding to ploidy levels: ‘Carousel Jolly Heart’ and ‘Carousel Pink Passion’ measured 2.06 pg and 2.05 pg, respectively, while ‘Sukim’ and ‘Freedom’ measured 4.18 pg and 4.27 pg, respectively.

## 1. Introduction

*Hibiscus* belongs to the Malvaceae family and comprises approximately 250 species of trees, shrubs, and herbs that grow throughout the world, including tropical, subtropical, and temperate regions [1]. Among them, *H. syriacus* (rose of Sharon or althea) is known as the national flower of Korea, with about 350 varieties which typically have a flowering period of 60–120 days [2]. Although its name suggests this species was first identified in Syria, *H. syriacus* likely originated in the Korean Peninsula and southern China and has since spread to Western countries [3]. Owing to their wide phenotypic variation, breeders most commonly focus on hybridizing *H. syriacus*. The cultivar *Hibiscus syriacus* ‘Sukim’ was developed in 2007 through hybridization between *H. sinosyriacus* ‘Malmauve’ and *H. syriacus* ‘Namwon’ and is characterized by a single-flower form [4]. It is an early maturing species that blooms from early July to late September. It was registered in 2009 with variety protection rights and is primarily planted as a street tree [4]. *H. syriacus* ‘Freedom’ was developed by Shadow Nursery Co. in the United States in 1997, with red-purple petals and a semi-double flower. Primarily, it is cultivated for ornamental gardens [5].

The *Hibiscus* Carousel series includes cultivars characterized by dark purple, deeply lobed foliage, abundant large flowers, a bushy growth habit, and low maintenance requirements. *H. moscheutos* ‘Carousel Jolly Heart’ and ‘Carousel Pink Passion’ are larger in size compared with *H. syriacus* and *H. sinosyriacus* [6]. These cultivars are pruned in spring and can withstand temperatures as low as −15 °C in winter. It can be grown in containers and thrives in full light and moist, nutrient-rich soils. Furthermore, they are suitable for border planting, mixed plantings, and mass plantings [6].

Understanding morphological and cytogenetic characteristics is essential for developing effective plant breeding methods. The basic chromosome number, total chromosome number, ploidy level, nuclear DNA contents, genomic sizes, and the locations of 5S and 18S (or 45S) rDNA loci are fundamental cytogenetic characteristics [7]. The basic chromosome number is one of the most important features for characterizing a taxonomic genus. Fluorescence in situ hybridization (FISH) and karyotyping are essential cytogenetic research techniques. FISH analysis provides fundamental information on a species’ ploidy level and chromosomal characteristics. Additionally, rDNA repeat sequences are commonly used as FISH probes to investigate the genetic relationships among different plant species [8]. Using FISH, oligos specific to a repetitive sequence or to a particular genomic region can be visualized [9]. Karyotype analysis is an essential cytological tool in plant breeding, enabling the identification and characterization of chromosomal abnormalities, ploidy levels, and structural variations that directly influence crop improvement strategies, though its application remains technically demanding in species with numerous small or morphologically similar chromosomes [10]. Genome size is an essential characteristic in systematic and evolutionary research [11]. Changes in DNA content and degree of ploidy are positively correlated. Genome size determination is helpful for elucidating relationships among species. So far, few studies on genome size or the nuclear DNA content of *Hibiscus* have been published [7,12]. Agro-morphological traits, including floral characteristics such as color, size, and type, as well as leaf shape and size, are among the most accessible and informative descriptors for evaluating cultivar diversity, supporting variety identification, and guiding selection in *Hibiscus* breeding programs. Although studies on *Hibiscus* highlight its practical importance, research that simultaneously evaluates both agro-morphological and cytogenetic traits remains limited. As a result, important gaps remain in understanding how observable characteristics are linked to the underlying genomic architecture. Since morphological variation in plants is frequently associated with differences in chromosome number, ploidy level, and genome organization, an integrated approach combining both types of analysis provide a more complete and reliable basis for cultivar characterization and interspecific comparison. *Hibiscus* genetic resources comprise extensive phenotypic and genomic diversity with significant implications for breeding and conservation. Integrated cytogenetic and morphological characterization provides a robust framework for facilitating interspecific hybridization, germplasm management, and ex situ conservation strategies [13,14].

Beyond their ornamental value, *Hibiscus* species collectively represent a rich reservoir of plant genetic resources with significance for food security, medicine, and ecological adaptation. *H. sabdariffa* (roselle) is cultivated across tropical Africa and Asia for its calyx, used in beverages, food colorants, and traditional medicine, while *H. cannabinus* (kenaf) is exploited as an industrial fiber crop. The genus thus spans a continuum from ornamental cultivars to economically important wild relatives and landraces, which are increasingly threatened by habitat loss, climate change, and the narrowing of cultivated gene pools [12,15]. Although this study focuses on cultivated *Hibiscus* cultivars, these represent a domesticated subset of a broader genetic continuum that includes wild relatives and traditional forms, which are critical reservoirs of adaptive diversity for conservation and crop improvement [16]. In this context, the comprehensive cytogenetic and morphological characterization of *Hibiscus* germplasm, including cultivated ornamentals with well-documented ploidy complexity, constitutes a foundational step toward structured genetic resource conservation. The cytogenetic baselines established for distinct accessions directly support germplasm registration, gene bank management, and the identification of genetically unique material for ex situ conservation, as advocated by current best practices in agrobiodiversity preservation [17,18]. Furthermore, ploidy variation in the genus, including the range from diploid to hexaploid across species [14,15], reflects evolutionary processes polyploidization, rDNA divergence, and genome size diversification that are directly relevant to understanding adaptation and resilience in the face of environmental change. Linking cultivated germplasm with wild genetic resources is essential to counteract genetic erosion and to support pre-breeding strategies under changing climatic conditions [19]. Accordingly, the present study aimed to comprehensively characterize selected *Hibiscus* cultivars through detailed assessment of floral traits (color, size, and type), leaf morphology (shape and size), and cytogenetic attributes, including chromosome number, karyomorphology, localization of 5S and 18S rDNA loci, nuclear DNA content, genome size, and ploidy level.

## 2. Results

### 2.1. Morphological Characteristics

**Figure 1:**
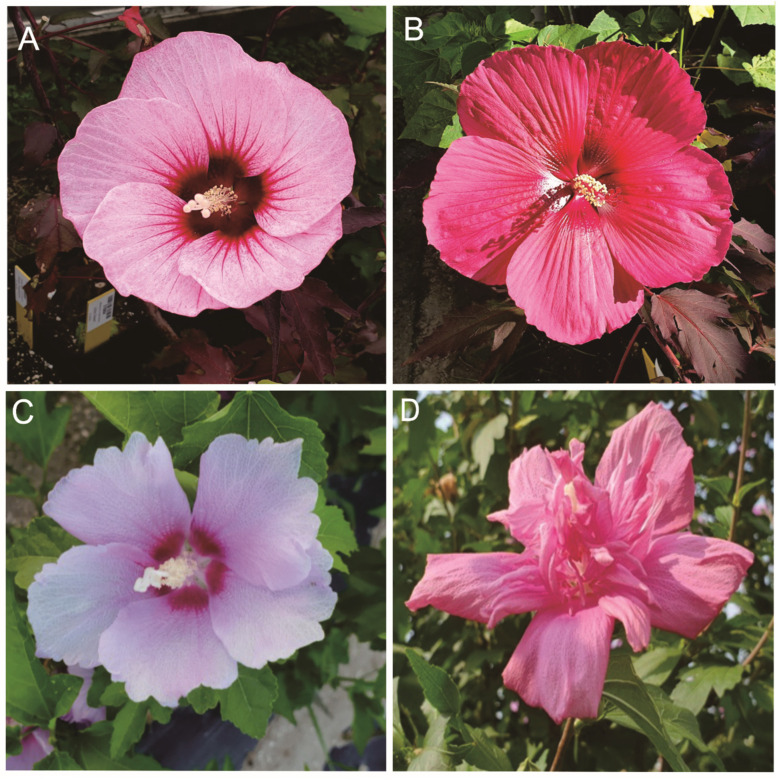
Flower morphology of *Hibiscus cultivars.* (**A**) *H. moscheutos* ‘Carousel Jolly Heart’; (**B**) *H. moscheutos* ‘Carousel Pink Passion’; (**C**) *H. syriacus* ‘Sukim’; (**D**) *H. syriacus* ‘Freedom’.

With regard to floral traits, *H. moscheutos* ‘Carousel Jolly Heart’ had pink flowers with a red center, and a flower diameter of 16.70 ± 0.36 cm (Figure 1A and Table 1). The length and width of the petal were 9.30 ± 0.84 cm and 10.4 ± 0.15 cm, respectively. Moreover, the length of the eye zone and length of the staminal column were 4.50 ± 0.09 cm and 4.30 ± 0.73 cm, respectively (Figure 1A and Table 1). The flower of *H. moscheutos* ‘Carousel Pink Passion’ was reddish pink, and the diameter of the flowers was 16.10 cm (Figure 1B and Table 1). The flower colors of *H. syriacus* ‘Sukim’ and ‘Freedom’ were light pink and red-purplish pink, respectively (Figure 1C,D and Table 1). The diameter of *H. syriacus* was substantially smaller than that of *H. moscheutos*: flower diameters of *H. syriacus* ‘Sukim’ (11.40 ± 0.45 cm) and ‘Freedom’ (10.20 ± 0.94 cm) were 30–38% smaller than those of ‘Carousel Jolly Heart’ (16.70 ± 0.36 cm) and ‘Carousel Pink Passion’ (16.10 ± 0.20 cm), respectively (Figure 1C,D and Table 1).

**Figure 2:**
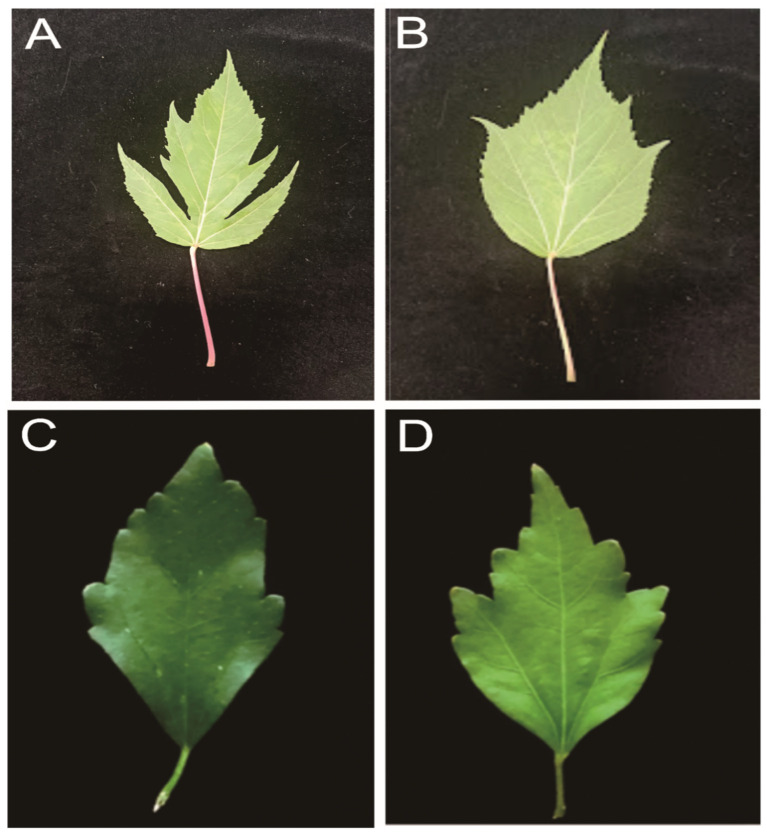
Leaf morphology of *Hibiscus* cultivars. (**A**) *H. moscheutos* ‘Carousel Jolly Heart’; (**B**) *H. moscheutos* ‘Carousel Pink Passion’; (**C**) *H. syriacus* ‘Sukim’; (**D**) *H. syriacus* ‘Freedom’.

Leaf shape is a key morphological characteristic. The leaf shapes of *H. moscheutos* ‘Carousel Jolly Heart’ and ‘Carousel Pink Passion’ were star-shaped and reniform, respectively, whereas *H. syriacus* ‘Sukim’ and ‘Freedom’ were elliptical in shape (Figure 2A–D). In this study, *H. moscheutos* ‘Carousel Jolly Heart’, ‘Carousel Pink Passion’, *H. syriacus* ‘Sukim’, and ‘Freedom’ had leaf lengths of 11.20 ± 0.94 cm, 12.53 ± 0.41 cm, 10.20 ± 0.38 cm, and 10.53 ± 0.41 cm, respectively (Figure 2A–D and Table 2).

### 2.2. Chromosome Counting

**Figure 3:**
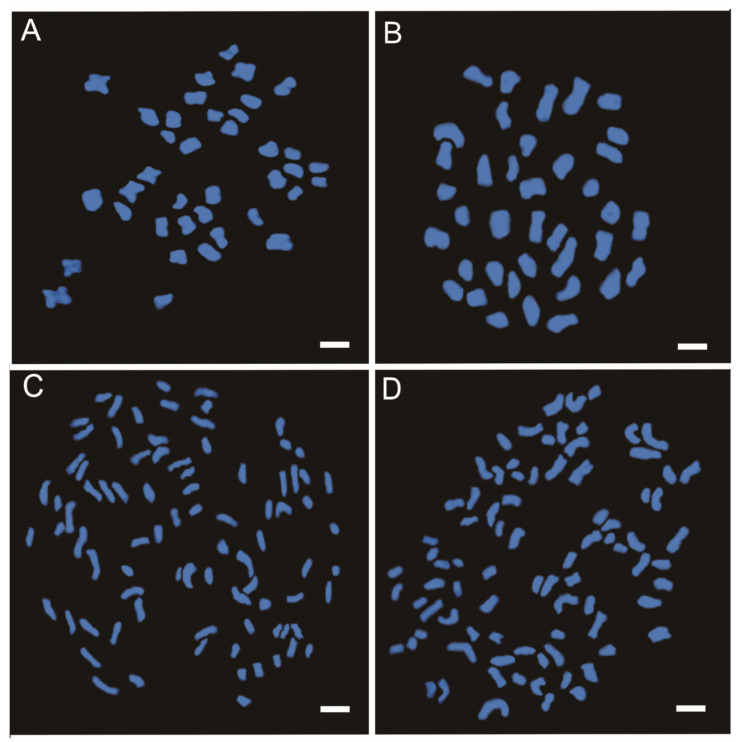
Chromosome number determination (**A**) *Hibiscus moscheutos* ‘Carousel Jolly Heart’ (2n = 38), (**B**) *Hibiscus moscheutos* ‘Carousel Pink Passion’ (2n = 38), (**C**) *Hibiscus syriacus* ‘Sukim’ (2n = 84) and (**D**) *Hibiscus syriacus* ‘Freedom’ (2n = 84). Viewed at 1000× Size bar 5 μm.

Five well-spread cells were photographed from each species to confirm the chromosome number. Thirty-eight chromosomes were confirmed in both cultivars of *H. moscheutos* (Figure 3A,B), whereas *H. syriacus* had eighty-four chromosomes (Figure 3C,D).

### 2.3. Chromosomal Localization of 5S rDNA and 18S rDNA Sites

**Figure 4:**
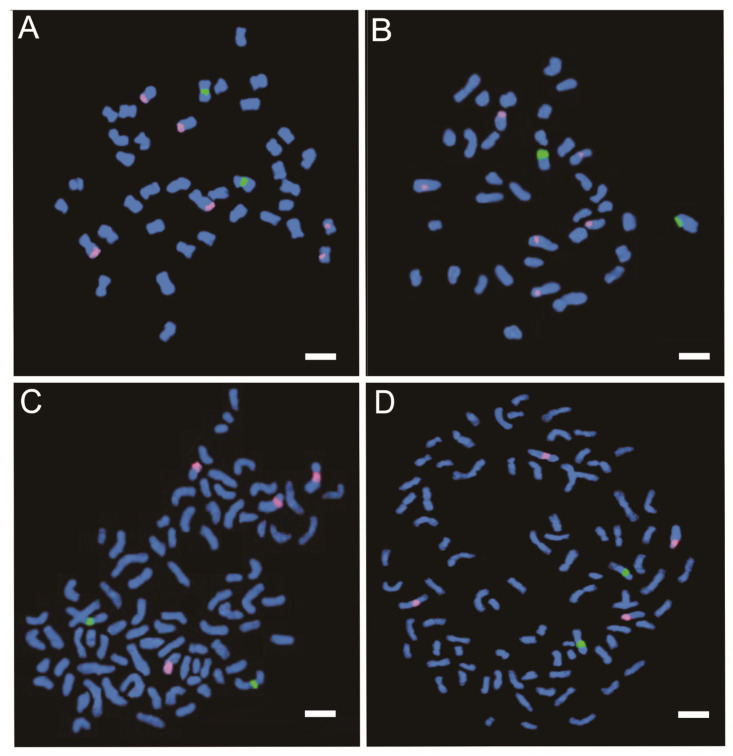
Distribution of 5S and 18S rDNA loci in *H. moscheutos* and *H. syriacus* revealed by FISH analysis. (**A**) *H. moscheutos* ‘Carousel Jolly Heart’; (**B**) *H. moscheutos* ‘Carousel Pink Passion’; (**C**) *H. syriacus* ‘Sukim’; (**D**) *H. syriacus* ‘Freedom’. Green signals indicate 5S rDNA loci (digoxigenin-labeled probe); red signals indicate 18S rDNA loci (biotin-labelled probe). Chromosomes are counterstained with DAPI (blue). Viewed at 1000× Size bar 5 μm.

Two 5S rDNA loci were found in both cultivars of *H. moscheutos* and *H. syriacus*. In addition, six 18S rDNA loci were detected in both cultivars of *H. moscheutos*, whereas four loci were observed in *H. syriacus* (Figure 4 and Table 3). The 5S rDNA loci in *H. moscheutos* ‘Carousel Pink Passion’ and ‘Carousel Jolly Heart’ were located at the centromeric region of chromosome # 7 and the short arm of chromosome #17, respectively. In contrast, the 5S rDNA loci in *H. syriacus* ‘Sukim’ and ‘Freedom’ were located on the long arm of chromosome # 9 and the short arm of chromosome #7, respectively (Figure 4A–D and Table 3).

The 18S rDNA loci were distributed at different chromosomal positions. In *H. moscheutos* ‘Carousel Pink Passion’, three pairs of 18S rDNA signals were detected: one pair on the long arm of chromosome #8, one pair on the short arm of chromosome #15, and one pair on the short arm of chromosome #19 (Figure 4B and Table 3). In *H. moscheutos* ‘Carousel Jolly Heart’, six 18S signals were detected, where one pair was detected in the long arms of chromosome 8, one pair was in the short arm of chromosome 18, and one pair was in the centromere position of chromosome 15 (Figure 4A and Table 3). In the case of *H. syriacus* ‘Sukim’, one pair of 18S rDNA signals was in the short arm of chromosome 19, and one pair in chromosome 22 (Figure 4C and Table 3). Two pairs of 18S rDNA signals were found in H. syriacus ‘Freedom’: one pair on the long arm of chromosome 33 and one pair on the short arm of chromosome 40 (Figure 4D and Table 3).

### 2.4. Karyomorphological Analysis

**Figure 5:**
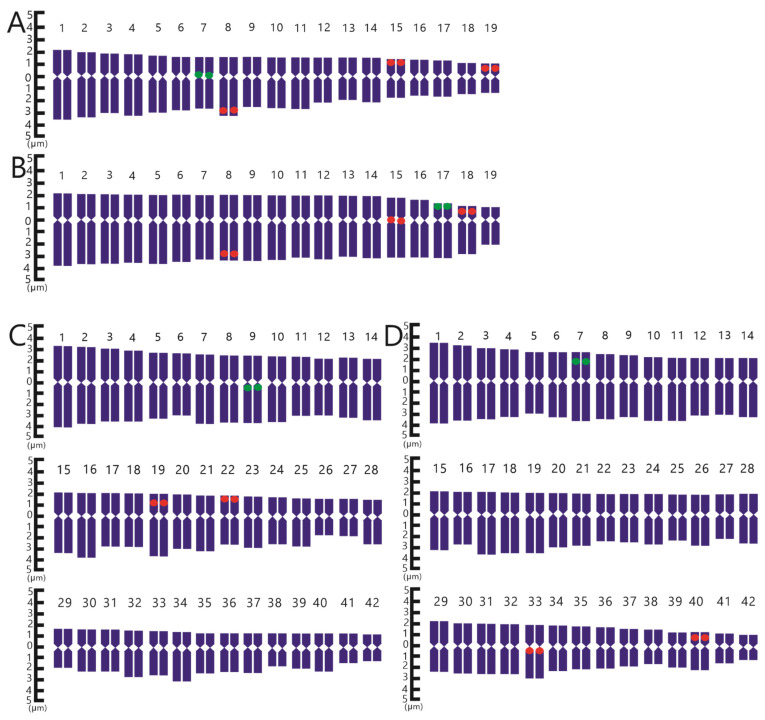
Karyotypes and ideograms show chromosomal localization of rDNA loci in *Hibiscus* cultivars. (**A**) *H. moscheutos* ‘Carousel Pink Passion’; (**B**) *H. moscheutos* ‘Carousel Jolly Heart’; (**C**) *H. syriacus* ‘Sukim’; (**D**) *H. syriacus* ‘Freedom’. Green signals indicate 5S rDNA loci (digoxigenin-labelled probe); red signals indicate 18S rDNA loci (biotin-labelled probe).

The chromosomes were arranged in decreasing order of short-arm length. An ideogram of the four cultivars is presented in Figure 5A–D and Table 4. The chromosome complement of *H. moscheutos* ‘Carousel Pink Passion’ comprised 38 chromosomes (Figure 5B and Table 4), with metaphase chromosome lengths ranging from 2.92 to 5.69 µm, and *H. moscheutos* ‘Carousel Jolly Heart’ was from 3.3 to 6.24 µm (Figure 5A and Table 4). The chromosome length of *H. syriacus* ‘Sukim’ ranged from 2.56 to 7.84 µm, and that of *H. syriacus* ‘Freedom’ ranged from 2.47 to 7.32 µm (Figure 5C,D and Table 4). Depending on the centromere positions, the homologous chromosomes are composed of metacentric and submetacentric pairs, and telocentric pairs were detected. 12 pairs of metacentric, 5 pairs of submetacentric, and 2 pairs of telocentric chromosomes were detected in *H. moscheutos* ‘Carousel Pink Passion; and *H. moscheutos* ‘Carousel Jolly Heart’ possessed 13 pairs of metacentric, 5 pairs of submetacentric, and 1 pair of telocentric chromosomes (Figure 5A,B and Table 4). In *H. syriacus* ‘Sukim’ and ‘Freedom’, the majority of chromosome pairs were metacentric, numbering 31 and 30, respectively, while 11 and 12 pairs were submetacentric, respectively (Figure 5C,D and Table 4).

### 2.5. Determination of Nuclear DNA Content

**Figure 6:**
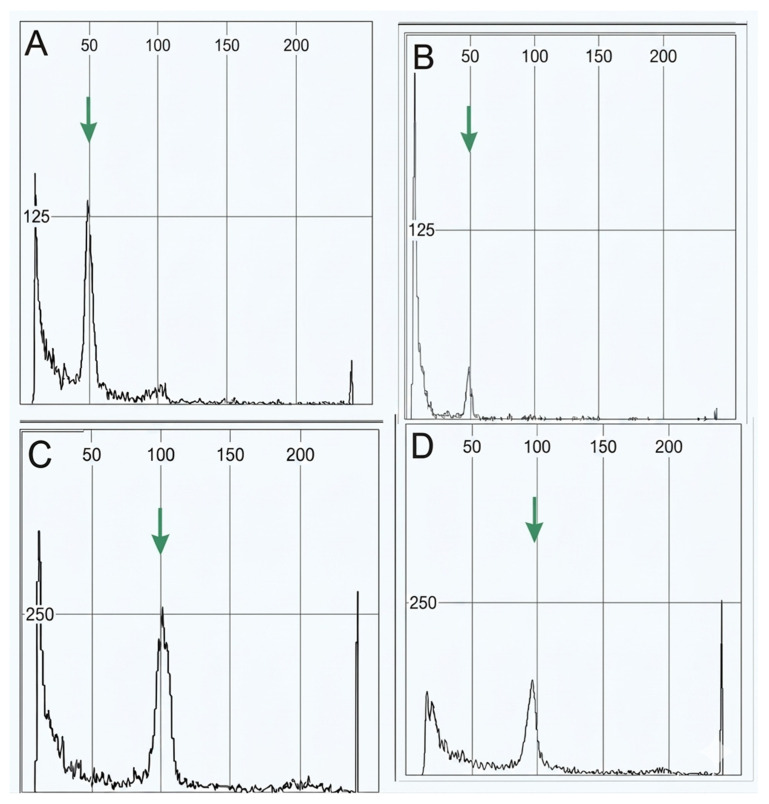
Flow cytometry histograms showing nuclear DNA content (2C values) of *Hibiscus* cultivars. (**A**) *H. moscheutos* ‘Carousel Pink Passion’; (**B**) *H. moscheutos* ‘Carousel Jolly Heart’; (**C**) *H. syriacus* ‘Sukim’; (**D**) *H. syriacus* ‘Freedom’. The arrow indicates the peak point.

Based on flow cytometry results, the 2C DNA contents of *H. moscheutos* ‘Carousel Pink Passion’ and ‘Carousel Jolly Heart’ were 2.06 pg and 2.05 pg, respectively (Figure 6A,B and Table 5). *H. syriacus* ‘Sukim’ and ‘Freedom’ had 2C DNA contents of 4.27 pg and 4.08 pg, respectively, which were approximately double those of *H. moscheutos* (Figure 6C,D and Table 5). The result of the 2C genome was 2016.71, 2004.9, 4176.06, and 3990.24 Mbp for *H. moscheutos* ‘Carousel Jolly Heart’ and ‘Carousel Pink Passion’ and *H. syriacus* ‘Sukim’ and ‘Freedom’, respectively (Figure 6A–D and Table 5).

## 3. Discussion

*Hibiscus* plants are cultivated in pots, gardens, and on street sides because of their abundant blooms and esthetic beauty, which require minimal work and expense. *H. moscheutos*, commonly known as swamp rose-mallow, is a perennial herbaceous plant that is indigenous to wetland regions in the eastern part of North America and can withstand cold temperatures throughout winter. The plant has been selectively developed for its large size and abundant, diverse flowers in different colors and shapes. Results showed diameters of *H. moscheutos* ‘Carousel Pink Passion’ and ‘Carousel Jolly Heart’ were 16.70 cm and 16.10 cm, respectively. In the experiments, we found that flower sizes in *H. moscheutus* are larger than those in *H. syriacus*, consistent with previous research [20]. *H. syriacus*, a winter-hardy, woody plant with flowers indigenous to Korea, has been officially designated as the national flower of Korea. The plant, commonly referred to as Rose of Sharon or Althea, has a flowering duration of 3–4 months [21]. Similarly, one study examined 127 different *Hibiscus* types and found that the *Hibiscus* hybrid ‘Daewangchun’ had the largest flower diameter, measuring 16.0 cm. On the other hand, *H. syriacus* ‘Ggoma’, ‘Mibeak’, ‘Andong’, and ‘Eunhasu’ had the smallest flowers, with each measuring approximately 6.2 cm. In the present study, the flower diameters *of H. syriacus* ‘Sukim’ and ‘Freedom’ were 11.40 cm and 10.20 cm, respectively [3].

From a genetic resource perspective, the evaluated cultivars constitute a representative subset of the broader *Hibiscus* germplasm, with direct relevance for conservation and breeding. Their characterization through integrated morphological and cytogenetic approaches aligns with current best practices, as no single method fully captures genetic diversity [7]. Morphological descriptors such as floral and leaf traits provide practical markers for cultivar identification, while cytogenetic parameters (chromosome number, karyotype structure, rDNA loci, and genome size) offer deeper resolution for germplasm classification. Notably, ploidy and genome size differences between ‘*H. moscheutos*’ (2n = 38; ~2.06 pg) and ‘*H. syriacus*’ (2n = 84; ~4.18–4.27 pg) reveal genomic constraints relevant to interspecific hybridization. These baselines inform strategies to overcome reproductive barriers [22,23] and underscore the need to maintain genetically distinct accessions within genebanks and ex situ collections. In an era of accelerating habitat loss and climate-driven range shifts, even ornamental germplasm with broad polyploid variation, such as *H. syriacus*, whose chromosomal numbers span eighty-ninety across cultivars, represents irreplaceable genetic diversity. The documentation of genome size, ploidy level, and rDNA locus configuration provided here thus constitutes a practical contribution to agrobiodiversity conservation, enabling precise accession characterization as a prerequisite for both germplasm registration and long-term safeguarding of *Hibiscus* diversity. In this context, well-characterized cultivars can serve as bridges between ex situ collections and wild gene pools, facilitating the introgression of adaptive traits into breeding programs [24].

However, cytogenetic information on these cultivars has not been reported to date, which is of great importance for breeding programs. Basic chromosome numbers are one of the key features of the cytogenetic characteristics of any genus. However, *Hibiscus* species have many small chromosomes, and the basic chromosome number of most species has not yet been determined. The range of basic chromosome numbers is wide: 7–44. Ploidy levels in this genus from diploid to sixteen-ploidy, and total chromosome number ranges from 22 to 180 [14,15]. In the experiment, 84 chromosomes were detected in *H. syriacus*, and 38 chromosomes were found in *H. moscheutos*, which is consistent with a previous result for *H. moscheutos* (38 chromosomes) [25]. However, some reported, published, and claimed that the chromosome number of *H. syriacus* is 80 [26]. Other reports have indicated that the chromosome number of *Hibiscus syriacus* ranges from 80 to 90, depending on the cultivar [25,27]. It was observed that there are over 150 cultivars of *H. syriacus*, with chromosomal numbers (80–90) and ploidy levels (diploid–octoploid) that are highly varied (unpublished data). There is a lot of variation in Old World lupins (*Lupinus*), not only for chromosomal counts (2n = 32–52), but also for the basic chromosome number (x = 5–9, 13) [28]. In the present study, the chromosome numbers of ‘Sukim’ and ‘Freedom’ are reported for the first time, and were determined to be 2n = 84. *H. moscheutos* was found to have a chromosome number of 38 (2n = 38), which is consistent with previously published reports [13,23,25]. Ref. [29] reported the following chromosome number in the genus *Hibiscus*: *H. schizopetalus* 2n = 42, *H. mutabilis* 2n = 92, *H. rosa-sinensis*, 2n = 4x = 84, *H. rosa-sinensis* “Double Rainbow” 2n = 5x = 105, *H. rosa-sinensis* ‘Flavo-plenus’ 2n = 6x = 138, and *H. rosa-sinensis* ‘Carminatus’ 2n = 7x = 147. Clearly, in the genus *Hibiscus*, chromosome numbers vary depending on ploidy and cultivar.

FISH is a molecular cytogenetic technique that uses fluorescent-labeled probes to classify complementary DNA sequences in nuclei [30]; the method has been shown to be technically efficient for cytogenetic studies of woody angiosperms [31]. The use of rDNA signals, combined with flow cytometry, has proved valuable for confirming ploidy levels in *Hibiscus*, a genus characterized by numerous small chromosomes and a high tolerance for polyploidy [14]. In our results, two 5S rDNA loci were in each *H. syriacus* ‘Sukim’ and ‘Freedom’ and *H. moscheutos* Carousel series. A study of 5S rDNA in cotton plants (close relatives to *Hibiscus*) revealed that most diploids had two 5S rDNA signals and all allotetraploid species had four 5S rDNA signals [32]. The same result was found in woody species of the genus *Rubus* [33]. The detection of only two 5S rDNA loci in *H. syriacus* and *H. moscheutos* may provide evidence of diploid status [3]. In addition, beyond inter-ploidy variation in rDNA loci, variation can also occur within the same ploidy level of a species. Reduced copy number and interstitial 5S rDNA are often observed in flowering plants [34]. Species with the same chromosome number have been found to have up to a five-fold difference in rDNA loci in a comparative analysis of species in *Brassicaceae* [35]. Our findings showed *H. moscheutos* and *H. syriacus* to be diploid based on cytogenetic results, according to previous studies [27,36].

Karyotyping is a useful tool for detecting chromosomal variation and is also valuable in the construction of genetic maps [37]. The karyotype is a primary cytological trait employed in both empirical and theoretical research [38]. In karyotype analysis, the difference within each species or cultivar may indicate changes in chromosome arrangement. In the present study, chromosome lengths of *H. moscheutos* ranged from 2.92 to 6.24 µm, whereas those of *H. syriacus* ranged from 2.47 to 7.84 µm. The chromosome length of *H. mutabilis f. mutabilis* was recorded as 1.24 to 10.89 µm which, was slightly higher than our studies [39]. Moreover, small chromosome studies such as chromosome length of the investigated *chrysanthemum* species were 9.70 to 12.24 µm, and 9.02 to 13.37 µm for *Chrysanthemum boreale* and *C. makinoi,* respectively [40]. The karyotype studies of *Populus* species (woody plants having small chromosomes) found that the relative chromosome length of *P. trichocarpa* is 4.30 to 11.02, and *P. euphratica* is 4.10 to 10.28 [41]. Based on centromere position, the chromosomes of *H. mutabilis* f. *mutabilis* can be categorized as metacentric or submetacentric, whereas those of *H. moscheutos* included metacentric (m), submetacentric (sm), and telocentric (st) types, and those of *H. syriacus* were classified as metacentric and submetacentric. Compared with simple shape classification, the application of karyotype data can address the difficulties of interpreting traditional chromosome shape and classification [42].

Flow cytometry provides a quick, precise, and straightforward method for determining the nuclear DNA content (C-value) of plants [43]. The technique facilitates the characterization of plant species in natural and agricultural settings, allows ready identification of ploidy levels, and is informative regarding the relationship between environmental conditions and evolutionary fitness. Despite the feasibility of flow cytometry methods, C-values have been calculated for only approximately 2% of the described angiosperm species [43]. The Plant DNA C-values database identified only one *Hibiscus* species with a recorded DNA C-value (*H. cannabinus*, 2C DNA = 3pg). In the present study, the 2C nuclear DNA content of *H. syriacus* ‘Sukim’ and *H. syriacus* ‘Freedom’ were found to be 4.27 pg and 4.08 pg, respectively, which were nearly equal to the cultivar of *H. syriacus* ‘Diana’, Lucy Woodbridge and Blue Bird (2C-DNA = 4.58, 4.62, 4.48, and 4.63 pg, respectively) [14,22]. *H. moscheutos* ‘Carousel Pink Passion’ had a nuclear DNA content of 2.06 pg, which was almost half that of tetraploid *Hibiscus syriacus* cultivars. The estimated genome size of *H. paramutabilis* (2C = 4.18 pg) was consistent with previously reported genome size values for tetraploid *H. sabdariffa* and *Gossypium* [1,44].

The cytogenetic diversity documented in this study, encompassing two distinct ploidy levels, contrasting rDNA locus configurations, and substantially different genome sizes between *H. moscheutos* and *H. syriacus*, has direct implications for the conservation of *Hibiscus* genetic resources under changing climatic conditions. Polyploidization is increasingly recognized as a mechanism conferring ecological plasticity and stress tolerance in plants [45,46], and the higher ploidy of *H. syriacus* (2n = 84; ~4.2 pg) relative to *H. moscheutos* (2n = 38; ~2.1 pg) may underlie differences in cold hardiness and adaptive potential relevant to climate change scenarios. The persistence of both diploid and polyploid cytotypes within the genus argues for their joint conservation as complementary components of *Hibiscus* genetic diversity. From a practical standpoint, the genomic baselines established here, chromosome number, karyotype structure, rDNA localization, and 2C DNA content, are precisely the parameters required for accession characterization in plant genetic resource programs [17,47], supporting gene bank registration, duplicate identification, and the monitoring of genetic integrity in ex situ collections over time. We therefore recommend that future germplasm surveys of *Hibiscus* explicitly incorporate cytogenetic markers alongside morphological descriptors to ensure comprehensive capture of the genetic diversity within this ornamentally and economically important genus.

## 4. Materials and Methods

### 4.1. Plant Materials

*Hibiscus moscheutos* ‘Carousel Jolly Heart’, ‘Carousel Pink Passion’, *Hibiscus syriacus*, ‘Sukim’ and ‘Freedom’ were used for this experiment. Stem cuttings of these plants were collected from the Korean National Arboretum and grown under field conditions, ensuring an appropriate environment at Kyungpook National University, Daegu, Republic of Korea. All plants used in the experiment were four years old.

Morphological characterization was performed on six plants per cultivar (n = 6) for each of the four *Hibiscus* cultivars studied: *H. moscheutos* ‘Carousel Jolly Heart’ and ‘Carousel Pink Passion’, and *H. syriacus* ‘Sukim’ and ‘Freedom’. Floral traits assessed included flower color, flower diameter, petal length and width, length of the eye zone, length of the staminal column, and flower type (single or semi-double). Leaf morphological traits recorded were leaf shape, leaf apex, leaf base, leaf color, leaf margin, leaf length, leaf width, diameter of the leaf shoulder, and petiole length. All quantitative measurements were performed using a digital caliper and recorded in centimeters. Data are presented as mean ± standard error (SE) of six individual plants per cultivar.

### 4.2. Phenotypic Characteristics

The morphological traits were explored using the Korean Seed & Variety Service’s (http://www.seed.go.kr) (accessed on 20 February 2022) survey method.

### 4.3. Root Collection and Chromosome Preparation

Root samples were collected at 07:00 from seedlings of *H. moscheutos* and *H. syriacus* and immediately placed in a light-protected tube containing a 2 mM solution of 8-hydroxyquinoline at room temperature for 4 h. Then, they were treated with a 3:1 (ethanol: acetic acid) solution for 24 h. Afterward, the root tips were washed with distilled water and preserved in 70% ethanol in −20 °C in a refrigerator until further use. The root tips were washed with distilled water to remove the fixative. Then, 0.7 to 1.0 mm segments of the distal root tip, where the actively dividing meristem is located, were excised, and 4–5 root meristems were transferred to 1.5 mL tubes with 60 µL of enzyme mixture containing 1% Cellulase, 1% Pectolyase (Seishin Pharmaceutical Co., Li, Hiratsuka, Japan) and 1% Cytohelicase in 0.01 M citrate buffer at 37 °C for 1 h. Digestion time may also vary depending on the size of the root tip, the age of the enzyme preparation, and other factors. Then, the tubes were removed from incubation and placed on ice for 5 min. The supernatant was carefully removed by pipetting to avoid disturbing the digested root meristems at the bottom of the tube. Carnoy’s solution (3:1) was added to the tubes and the treated roots. The tubes were vortexed briefly with digested root meristems to obtain a cell suspension, and the tubes were kept on ice for 5 min. Then, the tubes were centrifuged at 12,000 rpm for 2 min. Supernatant was discarded by pipetting. The pellet was resuspended in a 20–30 µL acetic acid:ethanol (9:1) solution. The slides were placed in a modified steam generator (i.e., a water bath at 80 °C) until the surface became granular (i.e., an ethanol meniscus formed on the surface of the slide: 10–15 s). Then, 10 µL of cell suspension was dropped onto the moistened slide. The slides were dried for further experiments.

### 4.4. Fluorescence In Situ Hybridization

The experiment was conducted using the FISH procedure described by [48] with minor modifications. The slides were treated with a solution consisting of 98 µg∙mL^−1^ RNase A and 2 mL 2× SSC buffer at a temperature of 37 °C for 45 min. Next, the slides were washed with 2× SSC buffer for 15 min, then rinsed with 4% paraformaldehyde for 10 min. The slides were washed with a series of ethanol solutions (70%, 90%, and 100%) for 9 min. Lastly, the slides were allowed to dry in the air.

Digoxigenin-labeled 5S rDNA (green fluorescence) and biotin-labelled 18S rDNA (red fluorescence) were mixed with formamide, 50% dextran sulfate, and 20× SSC to make a solution. Ensuring there were no bubbles, 40 µL of the mixture was added to each slide. For hybridization, slides were kept in a water bath adjusted to 82 °C for 5 min. Then, the slides were incubated for 45 min at 37 °C in the growth chamber. Again, the slides were washed in a water bath maintained at 42 °C while shaking in 0.1× SSC buffer for 30 min. Finally, ethanol series (70%, 90%, and 100%) was used to wash the slides.

4′,6-Diamidino-2-phenylindole (DAPI) and Vectashield mounting medium were mixed at a ratio of 2 parts DAPI to 100 parts Vectashield (Vector Laboratories, Burlingame, CA, USA) and used to stain the slides, which were then stored in the dark and examined under a microscope. The slides were analyzed using a Nikon BX-61 fluorescence microscope (Nikon, Tokyo, Japan). 5S and 18S rDNA signals were detected and captured using Cytovision 7.0 software.

### 4.5. Karyotype Analysis

For the karyotype analysis, five well-spread metaphase chromosomes were selected. The length of each chromosome was calculated using the Cytovision program (with a model Nikon BX 61 fluorescence microscope, Tokyo, Japan), and the chromosome number was estimated based on the short arm length [48]. Chromosome types were classified according to the ratio of the short arm to the long arm [49]. Images were captured using a CCD and then processed using the Genus image analysis workstation software (Genus version 3.8, Applied Imaging Corporation, Grand Rapids, MI, USA).

### 4.6. Determination of Nuclear DNA

Young leaves from the *Hibiscus* plants were used for flow cytometry. The analysis was performed using a Partec PA Ploidy Analyzer (Partec GmbH, Münster, Germany). The samples were sliced into 0.5 cm × 0.5 cm fragments and placed in a 50 × 12 mm Petri dish. They were then finely chopped using a blade. Subsequently, 500 μL of nucleus isolation buffer was introduced. Afterward, the crushed leaf sample was filtered into a tube to exclude any unwanted particles, and 2 mL of DAPI mixed staining buffer (Partech, GmbH, Munster, Germany) was introduced into the tube. Subsequently, the nucleus suspension was transferred into a flow cytometry analyzer. The calculation of 2C genome size involves multiplying the DNA content of a standard by the sample’s mean fluorescence, divided by the standard’s mean fluorescence [47]. *Raphanus sativus* ‘Saxa’, with a known 2C genome size of 1.10 pg, was used as an internal reference standard.

## 5. Conclusions

This study provides the first integrated cytogenetic and agro-morphological characterization of four *Hibiscus* cultivars representing two species of contrasting ploidy, contributing foundational data to the knowledge base for *Hibiscus* plant genetic resources. Distinct chromosomal features and rDNA distribution patterns were identified between the two species, providing fundamental insights into genome organization that are directly relevant to interspecific hybridization and trait-specific breeding in *Hibiscus*. The integrated characterization data generated in this study enrich the knowledge base for *Hibiscus* genetic resources and offer practical benchmarks for cultivar discrimination, germplasm registration, and gene bank management. Given the ornamental value and ecological significance of both species, the cytogenetic baselines established here also contribute to informed ex situ conservation strategies, ensuring that the phenotypic and genomic diversity represented by these cultivars is preserved for future breeding and restoration initiatives. Integrating such well-characterized cultivars with wild relatives and landraces will further strengthen conservation frameworks and enhance the sustainable use of *Hibiscus* genetic resources [50]. Collectively, these results affirm that systematic cytogenetic and morphological characterization is a prerequisite not only for the effective utilization of *Hibiscus* diversity in breeding programs but also for its long-term conservation, a priority that becomes increasingly urgent as climate change and habitat loss threaten the wild relatives and landraces that underpin the adaptive potential of this genus.

## Figures and Tables

**Table 1:** Morphological characteristics of *H. moscheutos* and *H. syriacus* (flower traits: color, diameter, petal dimensions, eye zone length, stamina column length, flower type).

Species	Flower Color	Flower Diameter (cm)	Petal Length (cm)	Petal Width (cm)	Length of Eye Zone (cm)	Length of Stamina Column (cm)	Flower Type
*H. moscheutos* ‘Carousel Jolly Heart’	Pink with red center	16.70 ± 0.36	9.30 ± 0.84	10.4 ± 0.15	4.50 ± 0.09	4.30 ± 0.73	Single
*H. moscheutos* ‘Carousel Pink Passion’	Reddish Pink	16.10 ± 0.20	8.10 ± 0.70	8.50 ± 0.52	3.20 ± 0.14	3.90 ± 0.77	Single
*H. syriacus* ‘Sukim’	Light pink	11.40 ± 0.45	5.90 ± 0.37	2.30 ± 0.17	2.90 ± 0.09	2.70 ± 0.29	Single
*H. syriacus* ‘Freedom’	Red-purplish pink	10.20 ± 0.94	4.90 ± 0.50	2.80 ± 0.06	2.30 ± 0.05	3.10 ± 0.10	Semi-double

Mean ± SE = 6.

**Table 2:** Leaf morphological characteristics of *H. moscheutos* and *H. syriacus*.

Species	Leaf Shape	Leaf Apex	Leaf Base	Leaf Color	Leaf Margin	Leaf Length (cm)	Leaf Width (cm)	Diameter of Leaf Shoulder (cm)	Petiole Length (cm)
*H. moscheutos* ‘Carousel Jolly Heart’	Star-shaped	Acuminate	Rounded	Light green	Serrate	11.20 ± 0.38	8.90 ± 0.21	8.32 ± 0.11	4.80 ± 0.10
*H. moscheutos* ‘Carousel Pink Passion’	Reniform	Acuminate	Rounded	Light green	Serrate	12.53 ± 0.41	9.13 ± 0.30	7.15 ± 0.18	4.50 ± 0.34
*H. syriacus* ‘Sukim’	Elliptical	Acute	Rounded	Dark green	Serrate	10.20 ± 0.38	8.20 ± 0.21	8.32 ± 0.11	3.80 ± 0.10
*H. syriacus* ‘Freedom’	Elliptical	Acute	Rounded	Green	Serrate	10.53 ± 0.41	9.13 ± 0.30	7.15 ± 0.18	3.50 ± 0.34

Mean ± SE = 6.

**Table 3:** Chromosomal localization of 5S rDNA and 18S rDNA sites in *H. moscheutos* and *H. syriacus*.

Species	No. of 5s rDNA	Location of 5S rDNA		Putative Chromosomes Pairs Containing 5S rDNA	No. of 18s rDNA	Location of 18s rDNA		Putative Chromosomes Pair Containing 18S rDNA
		**Long Arm**	**Short Arm**			Long Arm	Short Arm	
*H. moscheutos* ‘Carousel Jolly Heart’	2	0	2	Ch# 7	6	2	4	Ch# 8, 15, 19
*H. moscheutos* ‘Carousel Pink Passion’	2	0	2	Ch# 17	6	2	2 + 2	Ch# 8, 15,18
*H. syriacus* ‘Sukim’	2	2	0	Ch# 9	4	0	4	Ch# 19, 22
*H. syriacus* ‘Freedom’	2	0	2	Ch# 7	4	2	2	Ch# 33, 40

**Table 4:** Karyomorphological characteristics of *H. moscheutos* and *H. syriacus* (chromosome lengths and centromere position types).

Species	Longest Chromosome (µm)		Shortest Chromosome (µm)		No. of Chromosome Based on Centromere Position		
	Chr No	Chr Length	Chr No	Chr Length	Metacentric	Submetacentric	Telocentric
*H. moscheutos* ‘Carousel Pink Passion’	1	5.69	19	2.92	12	5	2
*H. moscheutos* ‘Carousel Jolly Heart’	1	6.24	19	3.3	13	5	1
*H. syriacus* ‘Sukim’	1	7.84	42	2.56	31	11	0
*H. syriacus* ‘Freedom’	1	7.32	41	2.47	30	12	0

**Table 5:** Nuclear DNA content, chromosome number, and ploidy level of *H. moscheutos* and *H. syriacus*.

Species	Putative DNA Content		Chromosome Number	Ploidy Level
	2C (Mbp)	2C (pg)		
*H. moscheutos* ‘Carousel Jolly Heart’	2016.71 ± 2.67	2.06 ± 0.05	38	2n = 2x = 38
*H. moscheutos* ‘Carousel Pink Passion’	2004.9 ± 2.63	2.05 ± 0.05	38	2n = 2x = 38
*H. syriacus* ‘Sukim’	4176.06 ± 5.61	4.27 ± 0.07	84	2n = 2x = 84
*H. syriacus* ‘Freedom’	3990.24 ± 5.63	4.08 ± 0.06	84	2n = 2x = 84

Mean ± SE = 6.

## Data Availability

The data presented in this study are available on request from the corresponding author. The data are not publicly available due to ongoing research activities.
